# Supplementary material for: miR-489-3p/SIX1 Axis Regulates Melanoma Proliferation and Glycolytic Potential
Source: Mol Ther Oncolytics. 2019 Nov 27;16:30–40. doi: 10.1016/j.omto.2019.11.001 (PMC7109510; doi:10.1016/j.omto.2019.11.001)
Supplement: Document S1. Figures S1–S5 [file mmc1.pdf]

OMTO, Volume *16*

Supplemental Information

miR-489-3p/SIX1 Axis Regulates Melanoma Proliferation and Glycolytic Potential

Xuhui Yang, Xiang Zhu, Zhifeng Yan, Chenxi Li, Hui Zhao, Luyuan Ma, Deyu Zhang, Juan Liu, Zihao Liu, Nan Du, Qinong Ye, and Xiaojie Xu

Figure S1

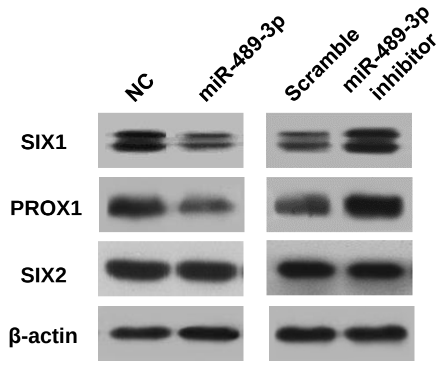

Supplementary Figure 1. Screening for the target of miR-489-3p in HEK293T cells. Immunoblot analysis of transfected with NC or miR-489-3p mimics, or scramble or miR-489-3p inhibitor in HEK293T cells . NC and scramble were used as negative control for miRNA mimics and miRNA inhibitor respectively. β–actin, a loading control.

Figure S2

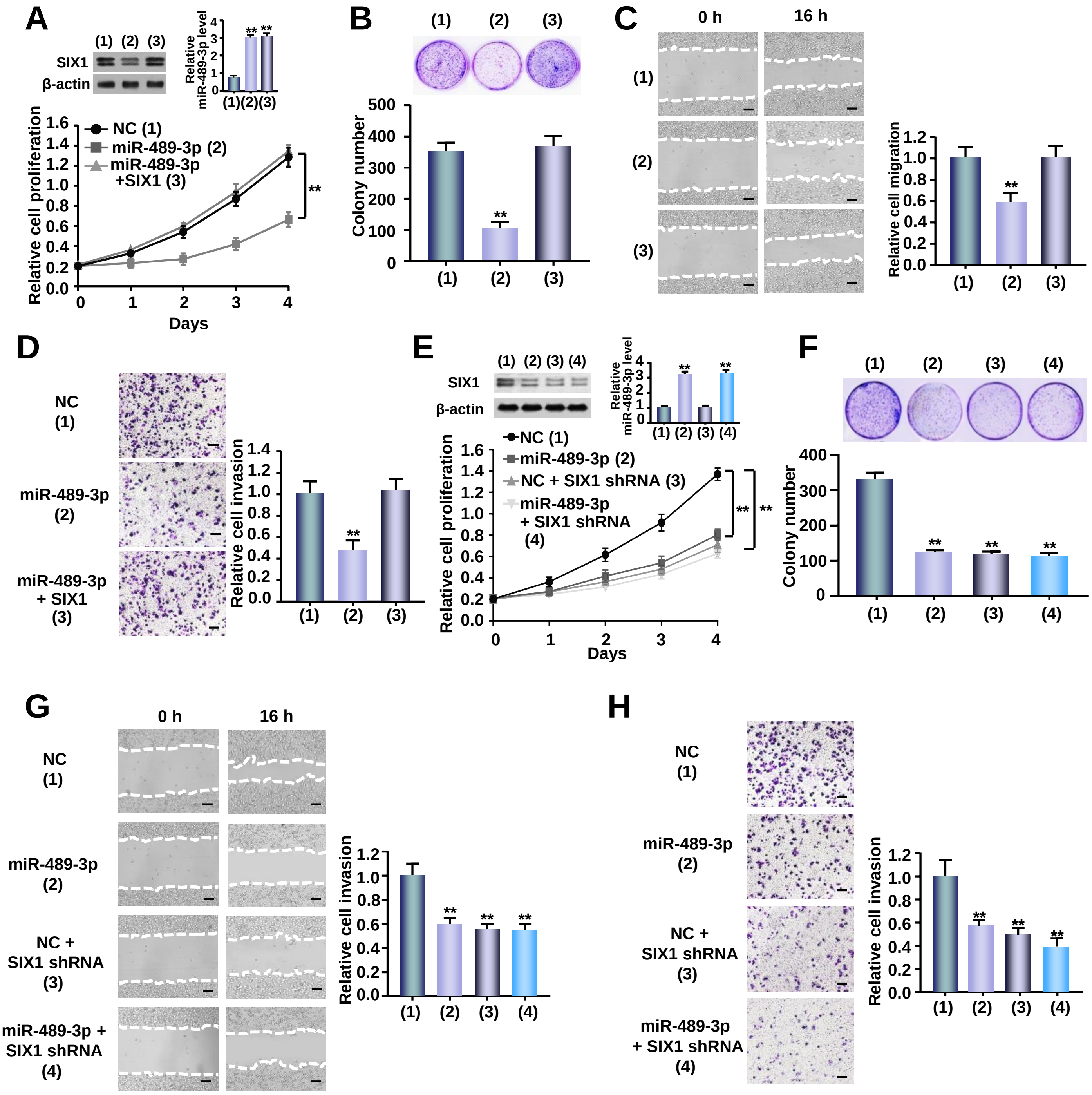

Supplementary Figure 2. miR-489-3p suppresses proliferation, migration and invasion via targeting SIX1 in SK-MEL-2 cells. (A) SK-MEL-2 cells were transfected with miR-489-3p mimics or miR-489-3p mimics plus SIX1 expression vector. Cell numbers was assessed by CCK-8 assay. The representative immunoblot shows SIX1 expression. Histograms display miR-489-3p expression determined by qRT-PCR. (B) Colony formation assay of SK-MEL-2 cells transfected as in (A). Illustrative images show colonies in plates (upper panels). Histograms show colony number. (C and D) Wound healing (C) and invasion (D) assays of SK-MEL-2 cells transfected as in (A). Right histograms show comparative cell migration and invasion. (E and F) Lentivirus mediated-SIX1 knockdown (SIX1 shRNA) or control SK-MEL-2 cells were transfected with NC or miR-489-3p mimics and analyzed as in (A) and (B). (G and H) Wound healing (G) and invasion (H) assays of lentivirus mediated-SIX1 knockdown (SIX1 shRNA) or control SK-MEL-2 cells were transfected as in (E). Scale bar, 100 μm. All values illustrated are mean ± SD of triplicate measurements and have been duplicated 3 times with similar results (**$p < 0.01$ versus corresponding control).

Figure S3

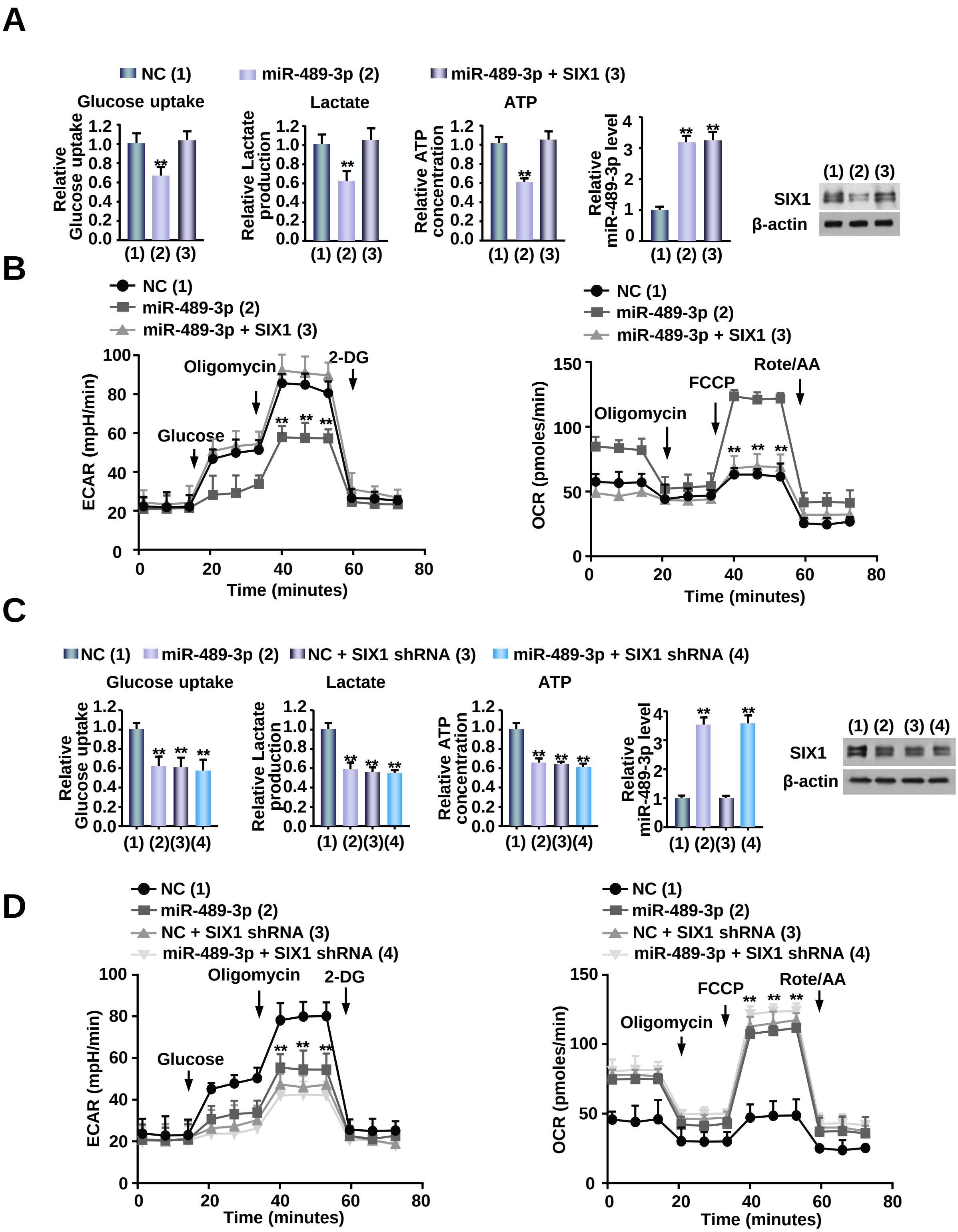

Supplementary Figure 3. miR-489-3p inhibits glycolysis via inhibition of SIX1 expression in SK-MEL-2 cells. (A) SK-MEL-2 cells were transfected with miR-489-3p mimics or miR-489-3p mimics plus SIX1. Glucose uptake and lactate production and ATP production were measured. Typical immunoblot reveals the expression of SIX1. qRT-PCR analysis shows miR-489-3p expression. (B) SK-MEL-2 cells were transfected as in (A), and ECAR and OCR were measured. (C) Lentivirus mediated-SIX1 knockdown (SIX1 shRNA) or control SK-MEL-2 cells were transfected with NC or miR-489-3p mimics and examined as in (A). (D) ECAR and OCR assays of lentivirus mediated-SIX1 knockdown (SIX1 shRNA) or control SK-MEL-2 cells were transfected as in (C). All values displayed are mean ± SD and have been duplicated 3 times with similar results (A-D). **$P < 0.01$ versus corresponding NC.

Figure S4

A

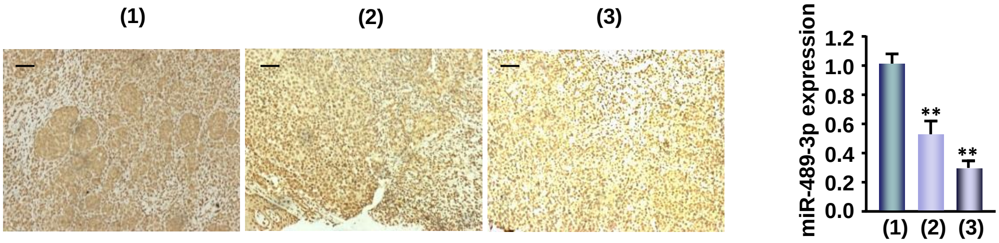

B

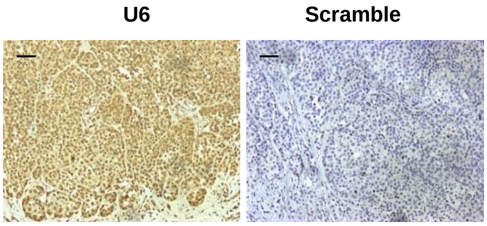

Supplementary Figure 4. Validation of the specificity of miR-489-3p probe. (A) Different miR-489-3p expression levels in 3 different melanoma tissues assessed by MISH (left panel) were measured by qRT-PCR (right panel). Data shown are mean ± SD of triplicate measurements and have been duplicated 3 times with similar results. ***P* < 0.01 versus case (1). (B) Positive control (U6) and negative control (Scramble) from the MISH kit were confirmed. Scale bar, 100 μm.

Figure S5

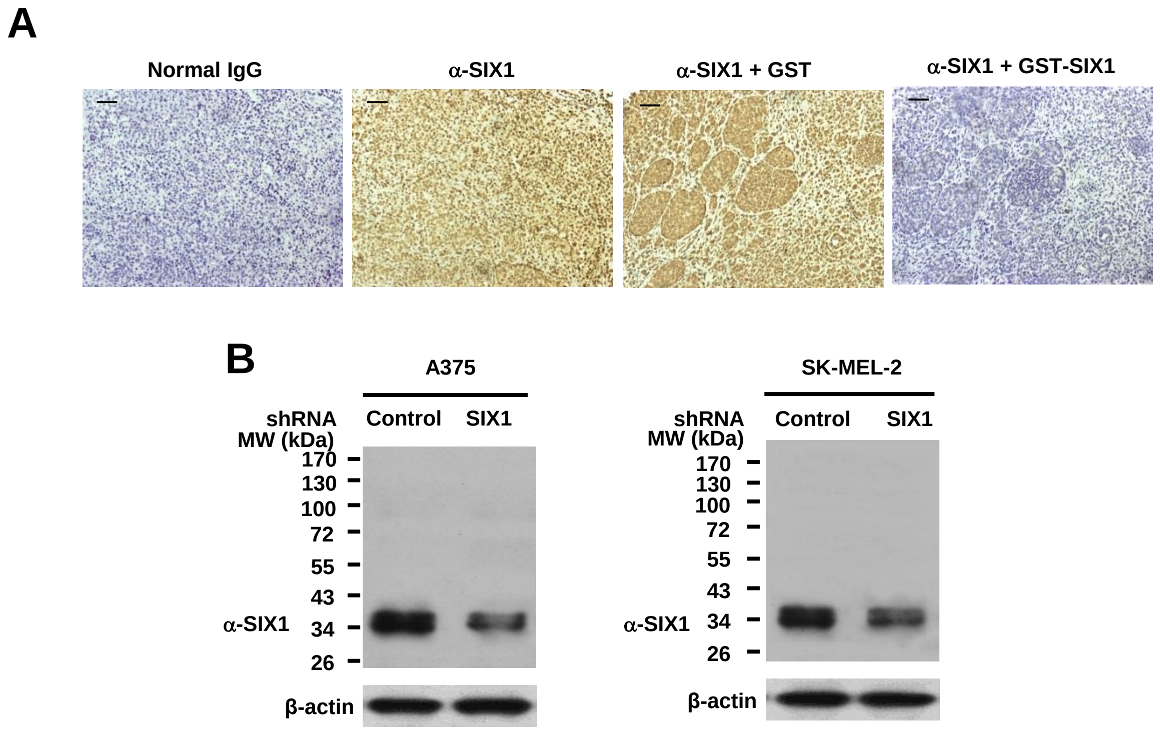

Supplementary Figure 5. Confirmation of antibody specificity to SIX1. (A) Immunohistochemical staining of melanoma samples incubated with normal IgG or anti-SIX1. To confirm antibody specificity, the anti-SIX1 was pre-incubated with recombinant GST-SIX1 protein or GST for 1 h prior to applying to tissue. Scale bar, 100 μm. (B) Immunoblot analysis of lysates from A375 (left panel) or SK-MEL-2 (right panel) cells infected with control shRNA or SIX1 shRNA using antibodies specific for anti-SIX1 β–actin, a loading control. MW, molecular weight.
