# Supplementary material for: miR-489-3p/SIX1 Axis Regulates Melanoma Proliferation and Glycolytic Potential
Source: Mol Ther Oncolytics. 2019 Nov 27;16:30–40. doi: 10.1016/j.omto.2019.11.001 (PMC7109510; doi:10.1016/j.omto.2019.11.001)
Supplement: Document S2. Article plus Supplemental Information [file mmc2.pdf]

Molecular Therapy
Oncolytics
Original Article

AMERICAN SOCIETY of GENE & CELL THERAPY

miR-489-3p/SIX1 Axis Regulates Melanoma Proliferation and Glycolytic Potential

Xuhui Yang,[1,2,4] Xiang Zhu,[1,4] Zhifeng Yan,[2,4] Chenxi Li,[2,4] Hui Zhao,[2] Luyuan Ma,[1] Deyu Zhang,[1] Juan Liu,[1] Zihao Liu,[2] Nan Du,[2] Qinong Ye,[1,3] and Xiaojie Xu[1]

[1]Department of Medical Molecular Biology, Beijing Institute of Biotechnology, No. 27 Taiping Road, Beijing 100850, China; [2]Department of Oncology, the 4th Medical Centre, PLA General Hospital, No. 51 Fucheng Road, Beijing 100191, China; [3]The First Affiliated Hospital, Zhengzhou University, Zhengzhou, Henan Province 450052, China

Sine oculis homeobox 1 (SIX1), a key transcription factor for regulating aerobic glycolysis, participates in the occurrence of various cancer types. However, the role of SIX1 in melanoma and the upstream regulating mechanisms of SIX1 remain to be further investigated. MicroRNAs (miRNAs) have emerged as key regulators in tumorigenesis and progression. Here, we show that miR-489-3p suppresses SIX1 expression by directly targeting its 3′ untranslated region (3′ UTR) in melanoma cells. miR-489-3p suppressed melanoma cell proliferation, migration, and invasion through inhibition of SIX1. Mechanistically, by targeting SIX1, miR-489-3p dampens glycolysis, with decreased glucose uptake, lactate production, ATP generation, and extracellular acidification rate (ECAR), as well as an increased oxygen consumption rate (OCR). Importantly, glycolysis regulated by the miR-489-3p/SIX1 axis is critical for its regulation of melanoma growth and metastasis both *in vitro* and *in vivo*. In melanoma patients, miR-489-3p expression is negatively correlated with SIX1 expression. In addition, patients who had increased glucose uptake in tumors and with metastasis assessed by positron emission tomography (PET) scans showed decreased miR-489-3p expression and increased expression of SIX1. Collectively, our study demonstrates the importance of the miR-489-3p/SIX1 axis in melanoma, which can be a potential and a promising therapeutic target in melanoma.

INTRODUCTION

Malignant melanoma, characterized by poor prognosis and rapid growth and metastasis, is a very aggressive and lethal form of cutaneous cancer.[1,2] Despite considerable advances in developing treatment options, understanding of the underlying mechanisms of melanoma growth and progression remains limited. Thus, it is essential to identify the biomarkers for melanoma, which is of great importance for improving survival outcomes and effective therapies.[3] Cancer cells frequently display high rates of aerobic glycolysis, one of the key mechanisms for cancer growth and progression.[4,5] Transcription factor plays a critical role in regulating the process of glycolysis in cancer cells. In our previous study, we have identified a key transcription factor, sine oculis homeobox 1 (SIX1), for regulation of the Warburg effect. We have found that SIX1 promotes aerobic glycolysis and is upregulated in various human cancers, such as breast infiltrative ductal carcinoma, liver cancer, and lung cancer. However, the role of SIX1 in melanoma remains largely unknown. Moreover, the upstream regulating mechanisms and the distinct functions that mediate SIX1 in melanoma remain to be further investigated. MicroRNAs (miRNAs) inhibit gene expression by preferentially binding to the 3′ untranslated regions (3′ UTRs) of target genes.[6] miRNAs regulate various processes in tumorigenesis and progression,[7] including cell proliferation, migration, and invasion. However, whether miRNA functions in melanoma via the process of aerobic glycolysis remains poorly characterized.

In this study, we show that miR-489-3p suppresses aerobic glycolysis in melanoma cells, resulting in inhibition of cancer cell proliferation, migration, invasion, and metastasis *in vitro* and *in vivo*. Mechanistically, SIX1 is a novel target of miR-489-3p. By directly targeting SIX1, miR-489-3p inhibits melanoma cell glycolysis, growth, and progression. In melanoma patients, miR-489-3p abundance is negatively correlated with SIX1 expression. Our data link the miR-489-3p/SIX1 axis to melanoma growth and progression, suggesting potential therapeutic strategies for melanoma.

RESULTS

miR-489-3p Inhibits SIX1 Expression by Targeting its 3′ UTR

To investigate the role of SIX1 in melanoma, we used two target prediction programs, TargetScan and miRanda, to screen for miRNAs that target SIX1. Our analysis predicted several potential SIX1-targeting miRNAs, among which only miR-489-3p and the positive control miR-548a-3p, which has been shown to inhibit SIX1 expression in breast and liver cancer cells,[8] could inhibit SIX1 expression in 293T cells by western blot analysis (Figure 1A). As previously reported,[9]

Received 26 March 2019; accepted 13 November 2019;
https://doi.org/10.1016/j.omto.2019.11.001.

[4]These authors contributed equally to this work.

Correspondence: Xiaojie Xu, Department of Medical Molecular Biology, Beijing Institute of Biotechnology, No. 27 Taiping Road, Beijing 100850, China.
E-mail: miraclexxj@126.com
Correspondence: Qinong Ye, Department of Medical Molecular Biology, Beijing Institute of Biotechnology, No. 27 Taiping Road, Beijing 100850, China.
E-mail: yeqn66@yahoo.com
Correspondence: Nan Du, Department of Oncology, the 4th Medical Centre, PLA General Hospital, No. 51 Fucheng Road, Beijing 100191, China.
E-mail: dunan05@aliyun.com

This is an open access article under the CC BY license (http://creativecommons.org/licenses/by/4.0/).

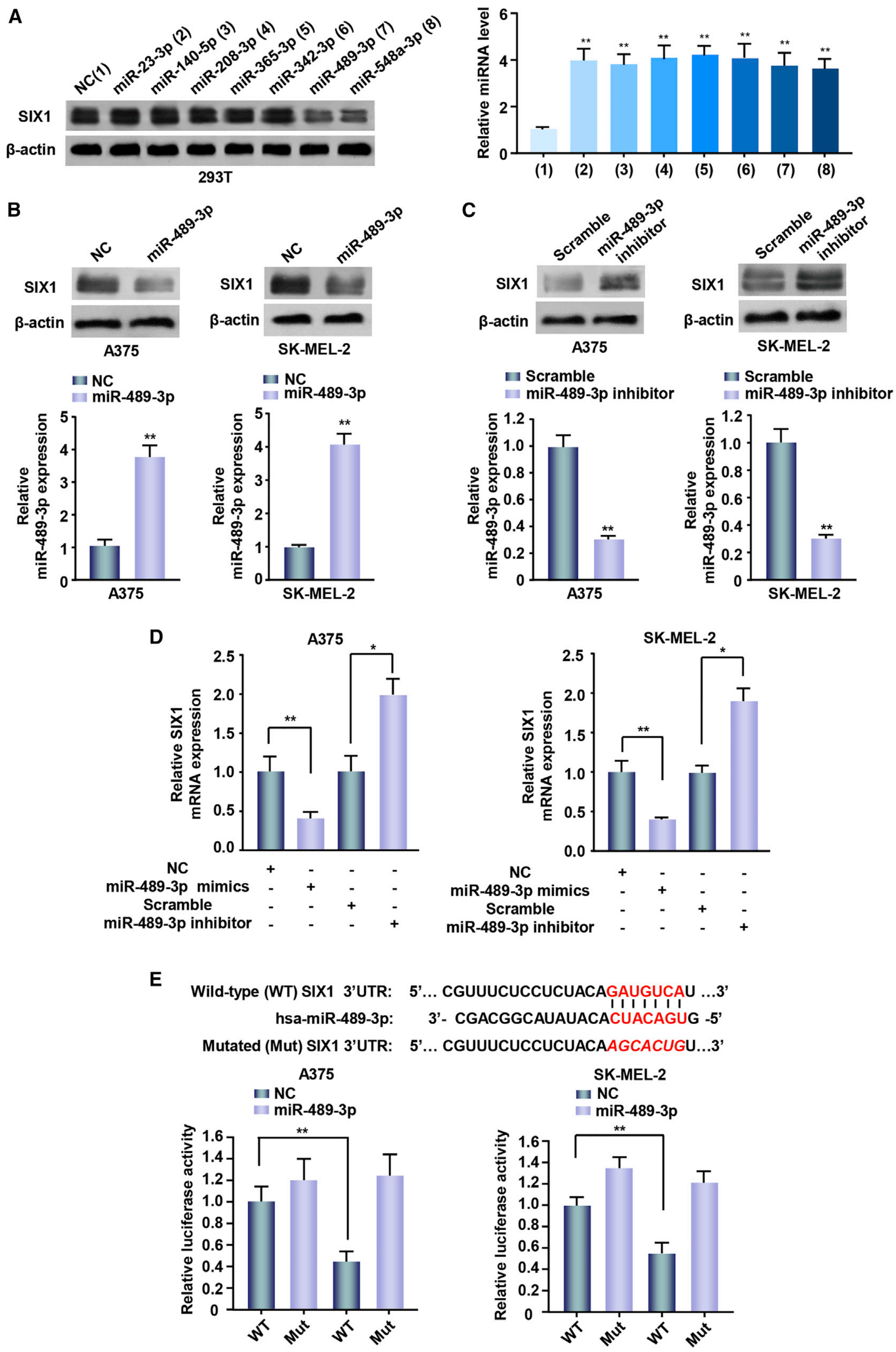

(legend on next page)

overexpression of miR-489-3p mimics inhibited prospero homeobox 1 (PROX1) expression (Figure S1). Moreover, miR-489-3p repressed the expression of SIX1, but not SIX2, another SIX family member. Therefore, we chose miR-489-3p for further study. Overexpression of miR-489-3p mimics inhibited SIX1 expression in the melanoma cell lines A375 and SK-MEL-2 (Figure 1B). In contrast, miR-489-3p inhibition upregulated SIX1 expression in the above-mentioned cell lines (Figure 1C). Quantitative reverse transcription PCR (qRT-PCR) further showed that miR-489-3p mimics decreased SIX1 mRNA expression while miR-489-3p inhibition increased SIX1 mRNA expression (Figure 1D).

To explore whether miR-489-3p is a direct and specific target of SIX1, we transfected A375 and SK-MEL-2 cells with wild-type SIX1 3′ UTR or 3′ UTR mutated luciferase reporter and miR-489-3p mimics. miR-489-3p decreased the SIX1 3′ UTR reporter activity, but not the luciferase activity of the mutant reporter in which the binding sites for miR-489-3p were mutated (Figure 1E). Taken together, these results suggest that miR-489-3p inhibits SIX1 expression by targeting its 3′ UTR in melanoma cells.

miR-489-3p/SIX1 Axis Mediates Melanoma Cell Proliferation, Migration, and Invasion

SIX1 has been shown to promote tumor cell proliferation, migration, and invasion. We tested if the miR-489-3p/SIX1 axis played similar functions in melanoma cells. Cell proliferation showed that overexpression of miR-489-3p mimics reduced the proliferation of A375 and SK-MEL-2 cells (Figure 2A; Figure S2A). Colony formation assays presented similar results in melanoma cells (Figure 2B; Figure S2B). These effects were reversed by SIX1 reexpression in the miR-489-3p-transfected cell lines. As expected, wound-healing and transwell assays demonstrated that miR-489-3p overexpression decreased migration and invasion ability (Figures 2C and 2D; Figures S2C and S2D). Conversely, SIX1 reexpression in the miR-489-3p-transfected cells reversed these effects. Moreover, SIX1 knockdown inhibited the ability of miR-489-3p to regulate these functions of melanoma cells (Figures 2E–2H; Figures S2E–S2H). Taken together, these results suggest that the miR-489-3p/SIX1 axis is critical for melanoma cell proliferation, migration, and invasion.

miR-489-3p Regulates Glycolysis via Inhibition of SIX1 Expression in Melanoma Cells

SIX1 is a key transcription factor involved in aerobic glycolysis. To confirm whether miR-489-3p might influence glycolysis via SIX1 in melanoma cells, we performed measurement of glucose uptake, lactate production, and ATP generation. As expected, miR-489-3p mimics decreased glucose uptake, lactate production, and ATP generation (Figure 3A; Figure S3A). Meanwhile, SIX1 reexpression in the miR-489-3p-transfected cells could reverse these effects. Additionally, both extracellular acidification rate (ECAR), reflecting overall glycolytic flux, and oxygen consumption rate (OCR), reflecting mitochondrial respiration, are indicators of glycolysis. The results showed that miR-489-3p mimics displayed decreased ECAR and increased OCR (Figure 3B; Figure S3B). Again, SIX1 reexpression in the miR-489-3p-transfected cells rescued these effects. miR-489-3p mimics in the SIX1 knockdown A375 and SK-MEL-2 cells had no effects on the glycolytic phenotype (Figures 3C and 3D; Figures S3C and S3D), indicating that miR-489-3p represses the glycolytic phenotype via SIX1. Taken together, these data collectively suggest that miR-489-3p dampens glycolysis via inhibition of SIX1 expression in melanoma cells.

miR-489-3p/SIX1 Axis Regulates Melanoma Growth, Metastasis, and Glycolysis *In Vivo*

To investigate the *in vivo* phenotype of the miR-489-3p/SIX1 pathway, A375 cells harboring miR-489 or SIX1 short hairpin RNA (shRNA) or miR-489 plus SIX1 shRNA were subcutaneously injected into the right flanks of male nude mice. Compared with the empty control vector, the tumors with miR-489 overexpression or SIX1 knockdown grew slowly (Figures 4A and 4B). Importantly, SIX1 knockdown abolished the ability of miR-489 to regulate the growth of cancer xenografts. Lactate production analysis of the tumor masses further validated that miR-489 significantly repressed the lactate production via SIX1 (Figure 4C). These data suggest that miR-489 suppresses tumor growth via SIX1-mediated glycolysis. In addition, the expression of Slug and Vimentin, the markers of epithelial-to-mesenchymal transition (EMT) involved in cancer metastasis, was downregulated by miR-489 overexpression or SIX1 knockdown, suggesting that the miR-489/SIX1 axis may play a role in metastasis (Figure 4D).

Meanwhile, we examined the effect of the miR-489/SIX1 axis on melanoma metastasis. The count of the luminescence spread of the pulmonary was significantly decreased in the miR-489-expressing group, compared with the empty vector group. Importantly, SIX1 knockdown almost abolished the ability of miR-489 to inhibit lung metastasis (Figure 4E). These data suggests that the miR-489/SIX1 axis may play a role in melanoma metastasis.

Figure 1. miR-489-3p Inhibits SIX1 Expression by Targeting Its 3′ UTR

(A) Immunoblot analysis of 293T cells transfected with negative control (NC, a universal negative control of RNA) or miR-23-3p, miR-140-5p, miR-208-3p, miR-365-3p, miR-342-3p, miR-489-3p, and miR-548a-3p mimics. Bar graphs display transfected miRNAs expression determined by qRT-PCR. (B and C) A375 and SK-MEL-2 cells were transfected with NC or miR-489-3p mimics (B) or scramble or miR-489-3p inhibitor (C). Scramble was a negative control for miRNA inhibitors. Bar graphs under the immunoblot reveal corresponding expression levels of transfected miRNAs testing by qRT-PCR. (D) RT-PCR analysis of SIX1 mRNA levels in the above melanoma cell lines transfected with the indicated constructs. (E) miRNA luciferase reporter assays of A375 and SK-MEL-2 cells transfected with wild-type or mutated SIX1 reporter and miR-489-3p mimics. Red font indicates the putative miR-489-3p binding sites within human SIX1 3′ UTR. Red and italicized font indicates mutated miR-489-3p binding sequences in the SIX1 3′ UTR (*$p < 0.05$, **$p < 0.01$). Each experiment was repeated at least twice, and one representative result was given. The data shown are the average values with error bars representing the SE (A–D).

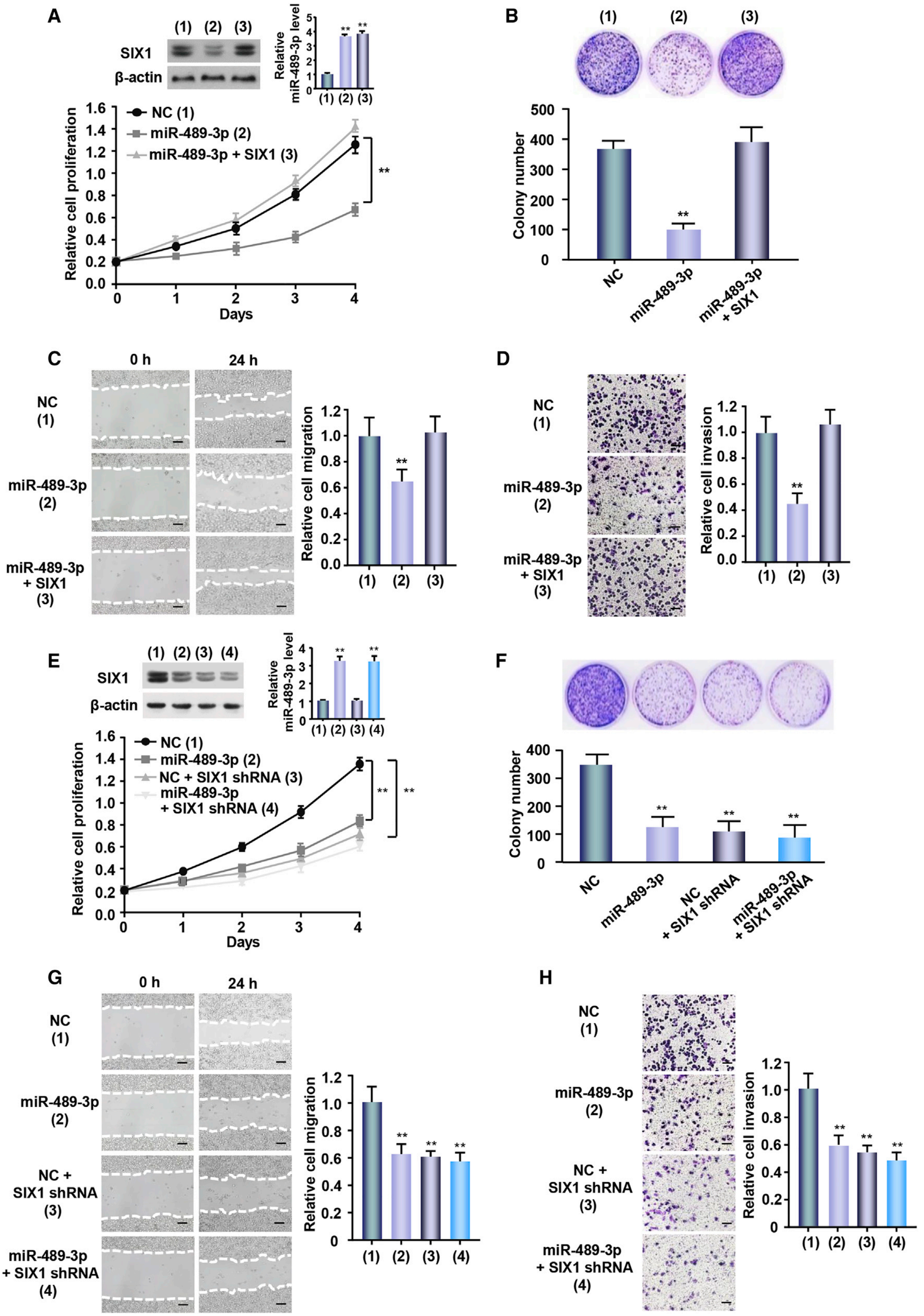

(legend on next page)

Correlation between miR-489-3p and SIX1 Expression and Correlation of miR-489-3p with Glucose Uptake in Human Melanoma Patients

We assessed SIX1 expression by immunohistochemical staining (IHC) and miR-489-3p expression by miRNA *in situ* hybridization (MISH) in 39 human melanoma samples. To confirm the cases, we utilized one of the melanoma indicators, S100, which is an effective marker for diagnosing and evaluating prognosis of melanoma patients.[10] In accordance with miR-489-3p inhibition of SIX1 in cultured cells, expression of SIX1 was inversely correlated with miR-489-3p expression in melanoma (Figures 5A and 5B). The association between miR-489-3p and SIX1 was further validated using external datasets from TCGA (The Cancer Genome Atlas) (Figure 5C). Interestingly, melanoma patients with increased glucose uptake and metastasis assessed by 2-^{18}fluoro-2-deoxy-D-glucose positron emission tomography (^{18}FDG PET) scans displayed decreased miR-489-3p expression and increased expression of SIX1 (Figure 5D). We confirmed the specificity of miR-489-3p staining by correlation analysis of miR-489-3p expression in melanoma tissues examined by MISH and qRT-PCR, respectively (Figure S4). The specificity of the SIX1 antibody was testified by IHC of melanoma tissues or immunoblotted with cell lysates (Figure S5). Taken together, these data suggest the miR-489-3p/SIX1 axis may be a promising way to treat melanoma patients.

DISCUSSION

SIX1 promotes proliferation, migration, invasion, and metastasis of multiple cancer cells, such as breast, liver, and gastric cancer cells. However, the role of SIX1 and the upstream regulators of SIX1 in melanoma are largely unknown. Our study confirmed the importance of the miR-489-3p/SIX1 axis in the Warburg effect and melanoma tumorigenesis and progression. miR-489-3p was proved as a novel SIX1-targeting miRNA in melanoma cells. miR-489-3p inhibits melanoma cell proliferation, invasion, and metastasis by directly targeting SIX1 expression by binding to its 3′ UTR. Mechanistically, the miR-489-3p/SIX1 axis regulates melanoma cell growth and metastasis by controlling glycolysis and mitochondrial respiration both *in vitro* and *in vivo*. Clinically, miR-489-3p is downregulated in melanoma patients and negatively correlated with SIX1, which is upregulated in melanoma patients. These findings indicate that the miR-489-3p/SIX1 axis may play an important role in the development and progression of melanoma (Figure 5E).

The SIX family, first characterized in *Drosophila*, is a group of transcription factors. Accumulating studies have showed that SIX1 participates in the occurrence of various human cancers. As a critical transcription factor, SIX1 plays a critical role in tumorigenesis.[11,12] Biological activity of SIX1 is controlled by several mechanisms. For example, SIX1 and eyes absent (EYA) are overexpressed, and the interaction between SIX1 and EYA occurred in multiple cancers.[13] EYA2 is required for the ability of SIX1 in mediating tumor progression.[14] SIX1 is a target gene controlled by WNT in acute myelocytic leukemia.[15] Recently, De Lope et al.[16] demonstrated that SIX1 promotes SOX2-mediated cellular plasticity during tumorigenesis. In endometrial carcinoma, SIX1 overexpression promotes cell growth through extracellular signal-regulated kinase (ERK)- and AKT-mediated pathways.[17] SIX1 promotes cell proliferation and tumorigenesis in osteosarcoma via regulating phosphatase and tensin homolog (PTEN)/ phosphoinositide-3-kinase (PI3K)/AKT signaling cascade.[18] In ovarian carcinoma, overexpression of SIX1 causes resistance to tumor necrosis factor-related apoptosis-inducing ligand (TRAIL)-mediated apoptosis.[19] In gastric cancer cells, SIX1 inhibits the mitochondrial apoptosis pathway via caspase-7, and it regulates proliferation and invasion by targeting the ERK pathway.[20,21] Liu et al.[22] showed that SIX1 promotes tumor lymphangiogenesis via coordinating transforming growth factor β (TGF-β) signals that increase expression of vascular endothelial growth factor C (VEGF-C). In breast cancer, SIX1 overexpression reinstates an embryonic pathway of proliferation by upregulating cyclin A1[21] and mediates resistance to paclitaxel in breast cancer cells.[22] Moreover, multiple studies have demonstrated that high SIX1 expression leads to poor survival in a variety of cancers.[23–25] In our concurrent study, in cultured melanoma cells, SIX1 was proven to promote cell proliferation and invasion, and SIX1 expression could be inhibited by miR-150-5p, which exhibited inhibitory functions in melanoma cell.[26] However, the *in vivo* metastatic functions and the clinical significance of SIX1 in melanoma are unknown. Thus, underlying the mechanisms of SIX1 regulation in melanoma and the upstream factors of SIX1 would be conducive to clinical treatments.

As is known, miRNAs are aberrantly expressed in pathological and physiological processes of many cancers. The miRNA pathway as a whole is a critical mechanism for gene expression control.[27] miRNAs exert their functions by targeting genes by binding their 3′ UTRs.[28] miRNAs are remarkably stable in the bloodstream. miRNA-based cancer gene therapy presents the possibility of targeting multiple gene networks mediated by miRNAs.[29] For example, in melanoma, miR-32 replacement therapy as a single agent has been demonstrated to suppress the growth of melanoma tumors in preclinical models via targeting myeloid cell leukemia 1 (MCL-1) and to exhibit synergistic

Figure 2. miR-489-3p/SIX1 Axis Regulates Proliferation, Migration, and Invasion in Melanoma Cells

(A) A CCK-8 assay was performed to detect relative proliferation compared to day 0 of the indicated groups of cells. The immunoblot shows SIX1 expression. Bar graphs indicate miR-489-3p expression performed by qRT-PCR. (B) A colony formation assay was performed in A375 cells transfected as in (A). Representative graphs show colonies in plates. Bar graphs show colony number. (C and D) Wound healing and Transwell invasion assays were performed in A375 cells transfected as in (A). Bar graphs show relative cell migration and invasion. (E-F) Lentivirus-mediated SIX1 knockdown (SIX1 shRNA) or control A375 cells were transfected with NC or miR-489-3p mimics. CCK-8 assay (E) and colony formation (F) were performed and analyzed. The immunoblot shows SIX1 expression. Bar graphs indicate miR-489-3p expression performed by qRT-PCR. (G-H) Lentivirus-mediated SIX1 knockdown (SIX1 shRNA) or control A375 cells were transfected with NC or miR-489-3p mimics. Wound healing (G) and Transwell invasion assays (H) were performed. Bar graphs show relative cell migration and invasion. Scale bars, 100 μm. (*$p < 0.05$, **$p < 0.01$ versus corresponding control). Each experiment was repeated at least twice and one representative result is given. The data shown are the average values with error bars representing the SE (A–H).

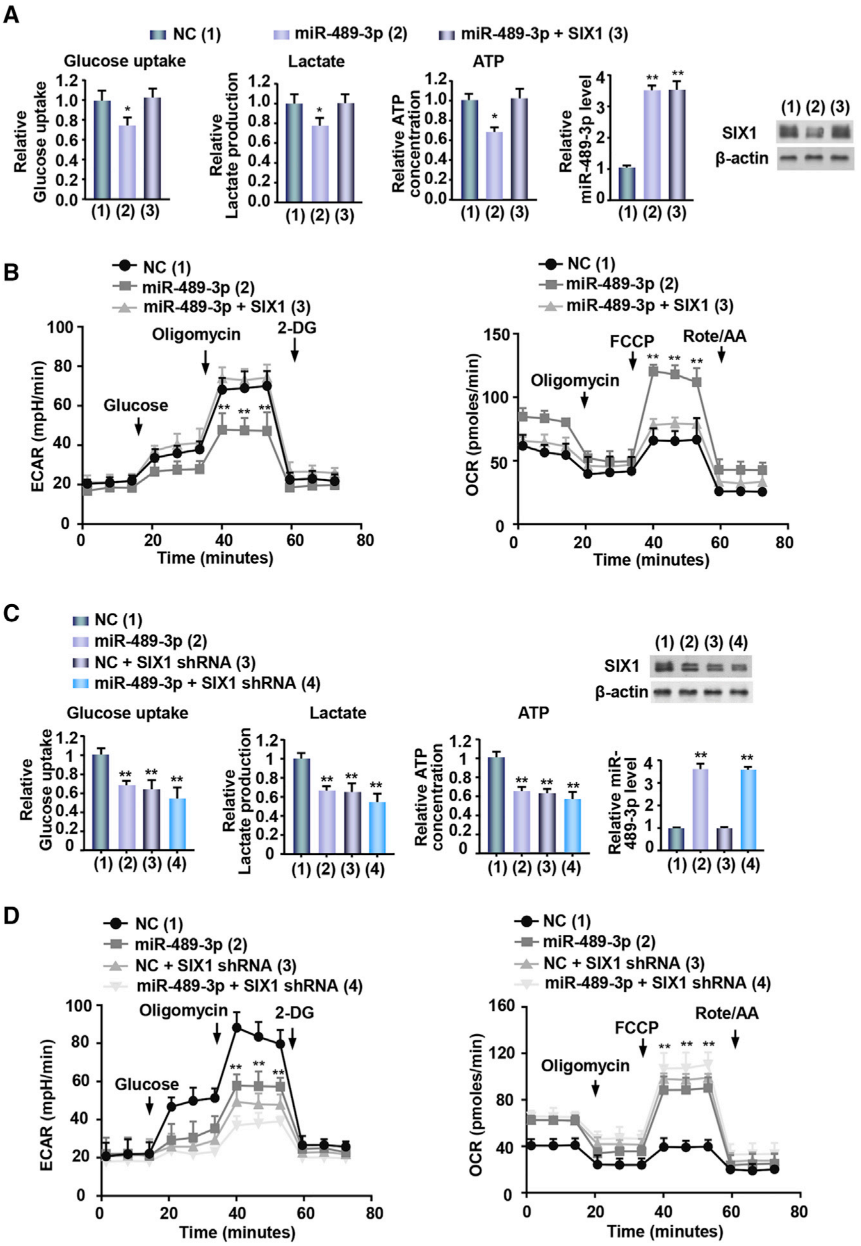

Figure 3. miR-489-3p/SIX1 Axis Regulates Glycolysis in Melanoma Cells

(A) A375 cells were transfected with miR-489-3p mimics or miR-489-3p mimics plus the SIX1 expression vector. Glucose uptake, lactate production, and ATP production were measured. Representative immunoblot reveals the expression of SIX1. qRT-PCR analysis indicates miR-489-3p expression. (B) A375 cells were transfected as in (A), and extracellular acidification rate (ECAR) and oxygen consumption rate (OCR) were determined. (C) Lentivirus-mediated SIX1 knockdown (SIX1 shRNA) or control A375 cells were transfected with NC or miR-489-3p mimics. Glucose uptake, lactate production, and ATP production were measured and analyzed. Representative immunoblot reveals the expression of SIX1. (D) A375 cells were transfected as in (C). ECAR and OCR were determined. All values shown are the average values with error bars representing the SE (*p < 0.05, **p < 0.01 versus corresponding control). Each experiment was carried out in triplicate, repeated at least twice, and one representative result is given (A–D).

effects with vemurafenib.[30] Recently, miR-489-3p has been found to be downregulated in various types of solid tumor, including colon cancer, breast cancer, lung cancer, and gastric cancer.[9,31–34] However, the role of miR-489-3p in melanoma remains unknown. The Warburg effect is one of the key mechanisms for cancer growth and metastasis. Glucose converts to pyruvate and then to the waste product lactic acid. It does not allow the mitochondria to oxidize pyruvate to CO_2 and H_2O in glycolysis. Glucose is converted into lactate without turning to mitochondria. A high level of aerobic glycolysis provides energy for cancer cell progression. In recent years, targeting the metabolism pathway has been proven to be a selective way to prevent tumor growth and metastasis and enhance anticancer treatments. In solid tumors, miR-489 has been identified as a tumor suppressor involved in increasing pathways such as human epidermal growth factor receptor-2 (HER2),[35,36] mitogen-activated protein kinase 1 (MEK1),[37] KRAS/nuclear factor κB (NF-κB)/Yin Yang 1 (YY1),[38] PI3K/AKT,[31] PROX1,[9] suppressor of zeste-12 (SUZ12),[33] protein-tyrosine phosphatases N11 (PTPN11),[39] histone deacetylase 7 (HDAC7),[32] and tumor protein, translationally controlled 1 (TPT1).[40] In melanoma, whether miR-489-3p is a key checkpoint of glycolysis remains to be further studied. We proved that miR-489-3p not only inhibits the Warburg effect in melanoma, but also suppresses melanoma growth and metastasis through inhibition of the SIX1-mediated Warburg effect.

In summary, we showed a novel mechanism that miR-489-3p regulates the transcription factor SIX1-mediated glycolysis in melanoma both *in vitro* and *in vivo*, The ability to target SIX1 by miR-489-3p elevates current understanding of the regulatory network of SIX1. The miR-489-3p/SIX1 axis may be a promising therapeutic target for melanoma.

MATERIALS AND METHODS

Cell Culture, RNA Oligonucleotides, and Reagents

Human malignant melanoma cell lines (A375, SK-MEL-2) were obtained from the American Type Culture Collection (Manassas, VA, USA). Cells were maintained in DMEM supplemented with 10% fetal bovine serum (FBS) at 37°C and 5% CO_2. Wild-type and mutated miR-489-3p putative targets on SIX1 3′ UTR were cloned into

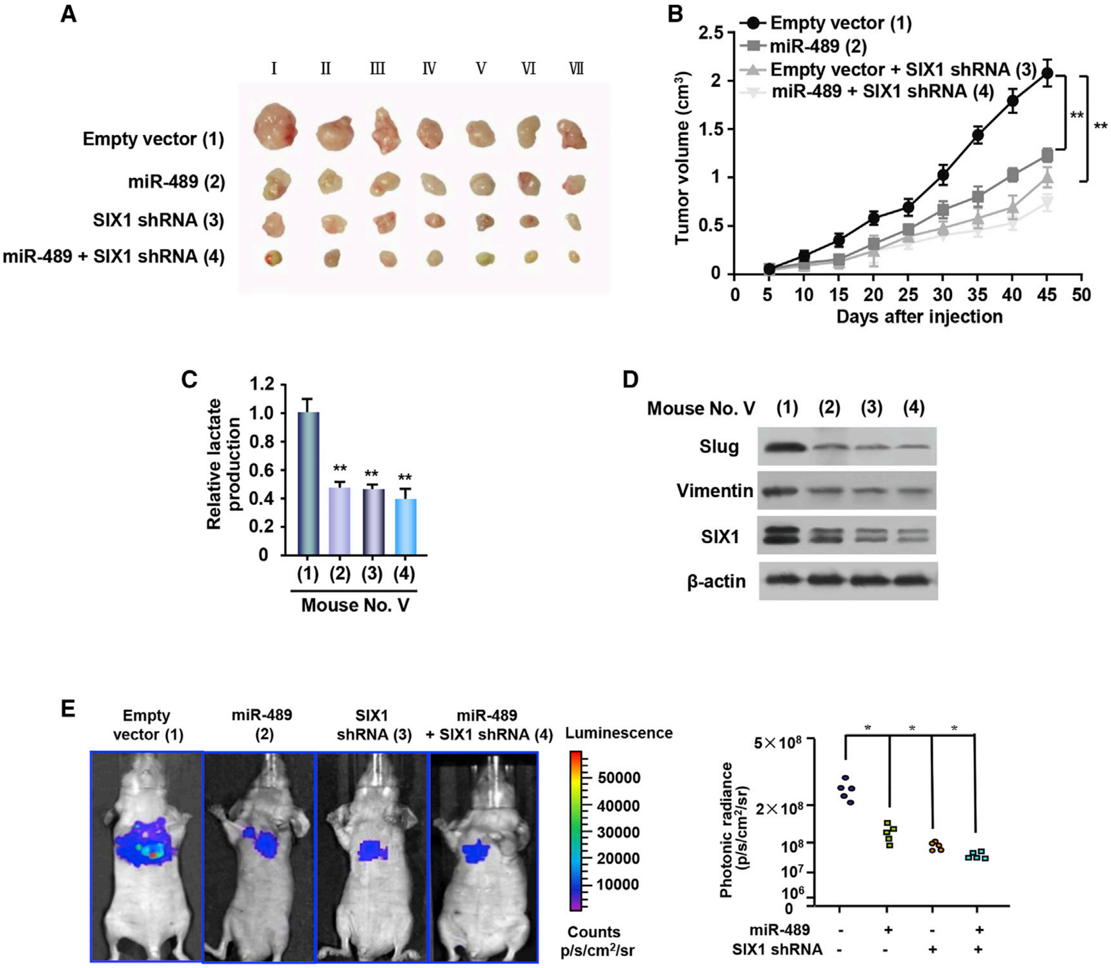

Figure 4. miR-489-3p/SIX1 Axis Regulates Glycolysis, Tumor Growth, and Metastasis *In Vivo*

(A) A375 cells stably infected with lentivirus carrying the indicated constructs were injected into nude mice as indicated. Roman numerals listed are mice. (B) At the indicated times, the tumors were measured (mean ± SE; n = 7), and the growth curves were plotted. **p < 0.01 at day 45. (C) Lactate production of representative tumor tissues from (A). All values shown are the average values of quintuplicate measurements with error bars representing the SE. **p < 0.01 versus empty vector. (D) Immunoblot analysis of the expressions of SIX1 and the EMT markers (Slug and Vimentin) in representative excised tumor from (A). (E) Melanoma cell metastases were established in nude mice (n = 5) by tail vein injection of A375 cells expressing indicated constructs. Bioluminescence images were collected at 30 days. Each experiment was repeated at least twice, and one representative result is given (A–E).

pmirGLO Dual-Luciferase miRNA target expression vector (Promega, USA). miR-489-3p mimics and miR-489-3p inhibitor were purchased from GenePharma (Shanghai, China). Anti-SIX1 was purchased from Proteintech Group (Rosemont, IL, USA), and anti-β-actin antibody was purchased from Santa Cruz Biotechnology (Dallas, TX, USA).

Cell Growth and Colony Formation Assays

Cell Counting Kit-8 (CCK-8) assays were performed to determine cell proliferation (Dojindo Laboratories, Kumamoto, Japan) following the producer's instructions. For the colony formation assay, transfected cells were plated in 3.5-cm plates (3,000 cells/well) as described previously.[41] After 2 weeks, colonies were fixed with 4% paraformaldehyde for 30 min and stained with 1% crystal violet for another 30 min. The number of colonies with diameters of more than 1.5 mm was counted.

Cell Migration and Invasion Assays

Cell migration was examined by wound healing assays. Transfected cells grown to 90% in six-well plates were scratched via a 200-μL pipette tip to create the wound followed by washing twice with PBS. Cultured cells were grown for 24 h to allow wound closure. The wound healing rates were calculated and compared to the width at 0 h. The cell invasion assay was performed with Matrigel invasion chambers following the producer's protocols (BD Biosciences). Transfected cells were seeded into the upper well. After 24 h, the invasive cells were fixed with 4% paraformaldehyde and stained with 0.5% crystal violet for 30 min, respectively. The number of

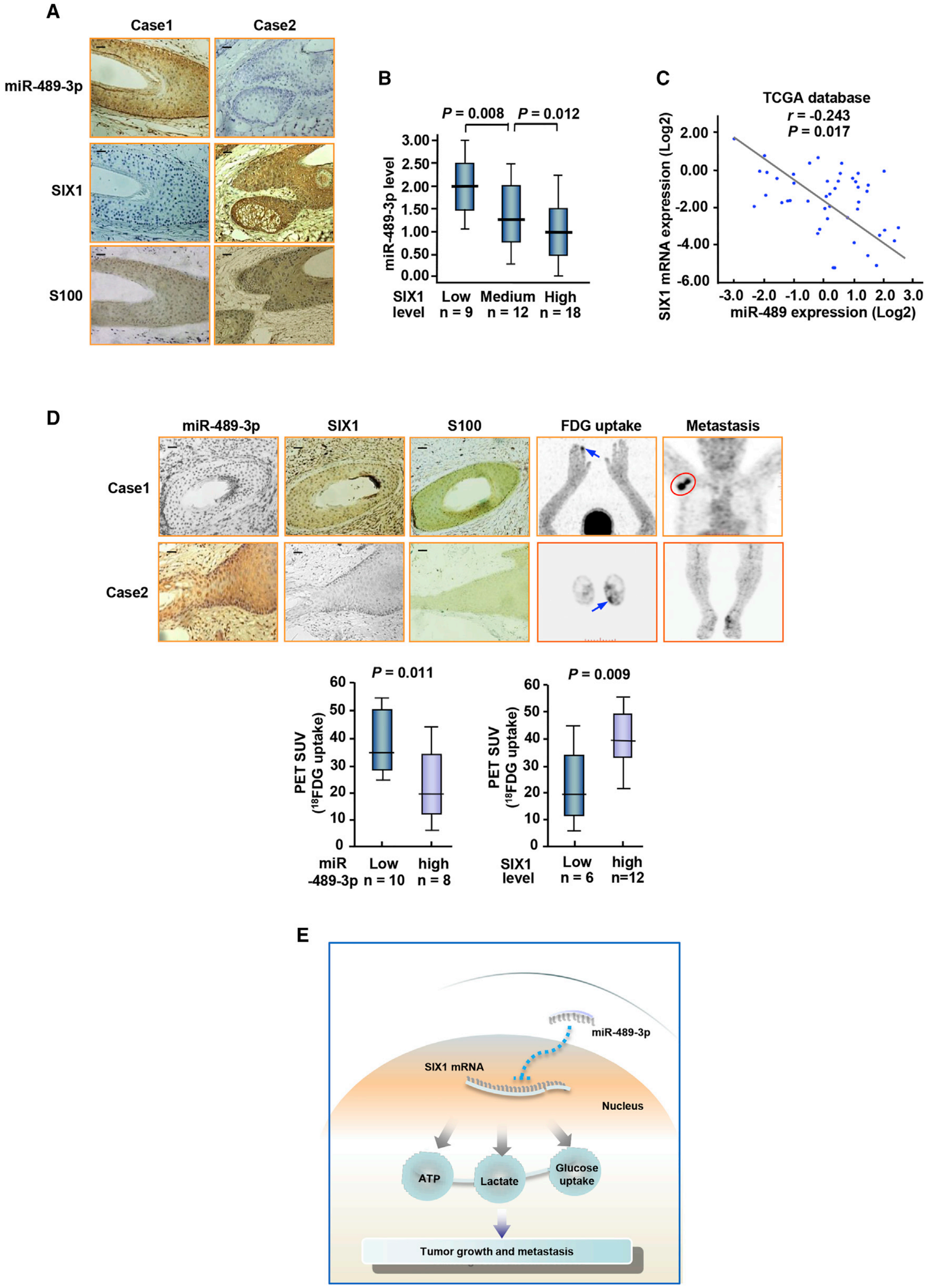

(legend on next page)

invasive cells was counted in casually selected microscope visions and imaged.

Luciferase Reporter Assay

1×10^5 cells per well were plated in 24-well plates. Cells were transiently transfected with luciferase reporters. Lipofectamine 2000 was used to cotransfect the wild-type or mutant SIX1 3′ UTR, in combination with miR-489-3p mimics or negative control for miRNA mimics. The cells were incubated for 48 h and then were harvested and analyzed for luciferase and β-galactosidase activities following the manufacturer's instructions (Promega, USA).

Measurement of Lactate, Glucose Uptake, and ATP

A glucose uptake colorimetric assay kit, lactate assay kit II, and ATP colorimetric assay kit were utilized following the manufacturer's instructions (BioVision, Milpitas, CA, USA). For glucose uptake, after cells were seeded, 100 μL of Krebs-Ringer-phosphate- *N*-2-hydroxyethylpiperazine-*N*′-2-ethanesulfonic acid (HEPES) buffer containing 2% BSA was added for 40 min, and then 10 mM 2-deoxy-D-glucose (2-DG) was added. For lactate and ATP assays, cells were homogenized in corresponding assay buffer offered by the kits and centrifuged at 4°C. For measurement of the lactate levels of mouse tumor, 10 mg of each tumor tissue was isolated and homogenized in the assay buffer (BioVision). The samples were centrifuged and the soluble fractions were measured and normalized to protein concentrations.

miRNA Extraction and qRT-PCR

A miRcute miRNA isolation kit (Tiangen) was used to extract the total RNA involving miRNA from cultured cells. Target miRNA was reverse transcribed to cDNA by the miRcute miRNA first-strand cDNA synthesis Kit (Tiangen). The expression level of miRNA was measured by a miScript SYBR Green PCR kit (QIAGEN, Venlo, the Netherlands) and performed on a CFX96 system (Bio-Rad, Hercules, CA, USA). The miR-489-3p expression level was assessed by qRT-PCR with the following primers: 5′-GGGGTGACATCACATATAC-3′ (forward) and 5′-CAGTGCGTGTCGTGGAGT-3′ (reverse). The control primers (U6) were 5′-CGCGCTTCGGCAGCACATATACT-3′ (forward) and 5′-ACGCTTCACGAATTTGCGTGTC-3′ (reverse). SIX1 mRNA expression was determined by qRT-PCR with the following primers: 5′-CGCGCACAATCCCTACCCATCGCC-3′ (forward), and 5′-CTTC CAGAGGAGAGAGTTGGTTCTG-3′ (reverse). The control primers for β-actin were 5′-ATCACCATTGGCAATGAGCG-3′ (forward) and 5′-TTGAAGGTAGTTTCGTGGAT-3′ (reverse).

ECAR and OCR Assays

ECAR and OCR were measured by a Seahorse XFe[96] extracellular flux analyzer (Seahorse Bioscience). Experiments were performed according to the manufacturer's protocol. 1×10^4 cells/well were plated in a Seahorse XFe[96] cell culture microplate. Data obtained were analyzed by Seahorse XFe Wave software.

Tumor Growth and Metastasis Analysis *In Vivo*

The animal study was approved and monitored by the Ethics Committees of the Chinese PLA General Hospital. For the *in vivo* tumor assay, a total of 1×10^7 A375 cells were subcutaneously inoculated into the right flank of nude mice. Tumor size was calculated at the indicated times. The mice were sacrificed at the indicated times. Excised tumors were conserved in liquid nitrogen.

For the lung metastasis study, 1×10^6 A375 cells labeled with firefly luciferase carrying indicated constructs were injected into the lateral tail vein of BALB/c mice. The animals were imaged on the day 30 using the IVIS200 imaging system (Xenogen, Alameda, CA, USA).

Immunohistochemistry and miRNA *In Situ* Hybridization

39 human melanoma samples were obtained from the Chinese PLA General Hospital, with the informed consent of patients and with approval for experiments. All patients were at the age of 25–75 years (mean age, 47 years). For patients used for ^{18}FDG PET scan analysis, MISH on paraffin tissue sections with probes specific for human miR-489-3p was performed following the manufacturer's protocols (Exonbio). Formalin-fixed, paraffin-embedded samples were performed as described previously.[42] Rabbit anti-SIX1 was as the primary antibody at dilutions of 1:100. In addition, the miR-489-3p and SIX1 scores were generated by multiplying the percentage of stained cells (0%–100%) by the intensity of the staining (low, 1+; medium, 2+; strong, 3+). Thus, the score is between 0 and 3. For correlation analysis, we defined a score <0.25, $0.25 \geq$ score < 0.75, and a score ≥ 0.75 as low, medium, and high SIX1. For PET scan analysis, we defined a score ≤ 0.75 and a score >0.75 as low and high SIX1 and miR-489-3p, respectively.

Statistical Analysis

Each experiment was performed in triplicate and repeated at least twice *in vitro*. The significance levels in cell proliferation, migration and invasion assays, glucose uptake, lactate, ATP, ECAR, and OCR,

Figure 5. Correlation between miR-489-3p and SIX1 and Correlation of miR-489-3p with Glucose Uptake in Human Melanoma Patients

(A) Representative IHC of 39 melanoma patients. SIX1 and S100 were determined by IHC and miR-489-3p by MISH. (B) The correlation of miR-489-3p with SIX1 in melanoma patients from (A) was analyzed. The low, medium, and high expression of SIX1 was determined as described in the Materials and Methods. Horizontal lines inside the box represent the median; the bottom and top of the boxes represent the 25th and 75th percentiles. The lines above and below the box represent the upper and lower extremes. The vertical bars represent the range of data. Data were analyzed by one-way ANOVA with Games-Howell correction. (C) Analysis of correlation of miR-489-3p expression with SIX1 mRNA using the data from The Cancer Genome Atlas (TCGA) (https://cancergenome.nih.gov/). (D) Representative FDG PET scans of two representative cases (case 1, melanoma with metastasis; case 2, melanoma with no metastasis) and IHC or miRNA *in situ* hybridization (MISH) of 18 melanoma patients. SIX1 and S100 were examined by IHC and miR-489-3p by MISH. Arrows reveal primary tumor glucose uptake. Red circle indicates the metastasis uptake. Scale bars, 100 μm. The correlation of glucose uptake with SIX1 or miR-489-3p expression was determined by the Mann-Whitney U test. (E) Proposed model for miR-489-3p modulation of SIX1 expression and subsequent regulation glycolysis-related tumor growth and metastasis.

as well as luciferase reporter assays, were assessed by a two-tailed Student's t test. The statistical analyses were computed by the SPSS 22.0 statistical software package. A p value of less than 0.05 was considered statistically significant. The correlation expression of miR-489-3p and SIX1 was examined by Spearman rank analysis.

SUPPLEMENTAL INFORMATION

Supplemental Information can be found online at https://doi.org/10.1016/j.omto.2019.11.001.

AUTHOR CONTRIBUTIONS

Conceptualization, X.X.; Formal Analysis, X.Z.; Funding Acquisition, X.X., Q.Y., and Z.Y.; Investigation, X.Y., X.Z., C.L., Z.L., and L.M.; Methodology, X.Y., X.Z., Z.Y., D.Z., and J.L.; Project Administration, N.D., X.X., and Q.Y.; Resources, Z.Y. and N.D.; Supervision, X.X., Q.Y., and N.D., Validation, X.Y., X.Z., Z.Y., and C.L.; Visualization, X.X. and X.Y.; Writing – Original Draft, X.X. and X.Y.; Writing – Review & Editing, X.X. and X.Y.

CONFLICTS OF INTEREST

The authors declare no competing interests.

ACKNOWLEDGMENTS

This work was supported by the National Natural Science Foundation of China (81822037, 81672602, 81972446, 81630067, 81872246, 81502264, and 81872090); the Logistics Scientific Research project (BWS16J010); the National Key Research and Development Program of China (2017YFA0505602); and Capital Foundation of Medical Developments (2016-4-5061).

REFERENCES

1. Rastrelli, M., Tropea, S., Rossi, C.R., and Alaibac, M. (2014). Melanoma: epidemiology, risk factors, pathogenesis, diagnosis and classification. In Vivo *28*, 1005–1011.
2. Trotter, S.C., Sroa, N., Winkelmann, R.R., Olencki, T., and Bechtel, M. (2013). A global review of melanoma follow-up guidelines. J. Clin. Aesthet. Dermatol. *6*, 18–26.
3. Shtivelman, E., Davies, M.Q., Hwu, P., Yang, J., Lotem, M., Oren, M., Flaherty, K.T., and Fisher, D.E. (2014). Pathways and therapeutic targets in melanoma. Oncotarget *5*, 1701–1752.
4. Liberti, M.V., and Locasale, J.W. (2016). The Warburg effect: how does it benefit cancer cells? Trends Biochem. Sci. *41*, 211–218.
5. Potter, M., Newport, E., and Morten, K.J. (2016). The Warburg effect: 80 years on. Biochem. Soc. Trans. *44*, 1499–1505.
6. Ambros, V. (2004). The functions of animal microRNAs. Nature *431*, 350–355.
7. Berindan-Neagoe, I., Monroig, Pdel.C., Pasculli, B., and Calin, G.A. (2014). MicroRNAome genome: a treasure for cancer diagnosis and therapy. CA Cancer J. Clin. *64*, 311–336.
8. Yang, Z., Zhang, T., Wang, Q., and Gao, H. (2018). Overexpression of microRNA-34a attenuates proliferation and induces apoptosis in pituitary adenoma cells via SOX7. Mol. Ther. Oncolytics *10*, 40–47.
9. Zhang, H., Xie, X., Zhu, X., Zhu, J., Hao, C., Lu, Q., Ding, L., Liu, Y., Zhou, L., Liu, Y., et al. (2005). Stimulatory cross-talk between NFAT3 and estrogen receptor in breast cancer cells. J. Biol. Chem. *280*, 43188–43197.
10. Li, L., Liang, Y., Kang, L., Liu, Y., Gao, S., Chen, S., Li, Y., You, W., Dong, Q., Hong, T., et al. (2018). Transcriptional regulation of the Warburg effect in cancer by SIX1. Cancer Cell *33*, 368–385.e7.
11. Zhang, B., Ji, S., Ma, F., Ma, Q., Lu, X., and Chen, X. (2016). miR-489 acts as a tumor suppressor in human gastric cancer by targeting PROX1. Am. J. Cancer Res. *6*, 2021–2030.
12. Salama, I., Malone, P.S., Mihaimeed, F., and Jones, J.L. (2008). A review of the S100 proteins in cancer. Eur. J. Surg. Oncol. *34*, 357–364.
13. Wu, W., Ren, Z., Li, P., Yu, D., Chen, J., Huang, R., and Liu, H. (2015). Six1: a critical transcription factor in tumorigenesis. Int. J. Cancer *136*, 1245–1253.
14. Zheng, W., Huang, L., Wei, Z.B., Silvius, D., Tang, B., and Xu, P.X. (2003). The role of *Six1* in mammalian auditory system development. Development *130*, 3989–4000.
15. Kingsbury, T.J., Kim, M., and Civin, C.I. (2019). Regulation of cancer stem cell properties by SIX1, a member of the PAX-SIX-EYA-DACH network. Adv. Cancer Res. *141*, 1–42.
16. De Lope, C., Martín-Alonso, S., Auzmendi-Iriarte, J., Escudero, C., Mulet, I., Larrasa-Alonso, J., López-Antona, I., Matheu, A., and Palmero, I. (2019). SIX1 represses senescence and promotes SOX2-mediated cellular plasticity during tumorigenesis. Sci. Rep. *9*, 1412.
17. Xin, X., Li, Y., and Yang, X. (2016). SIX1 is overexpressed in endometrial carcinoma and promotes the malignant behavior of cancer cells through ERK and AKT signaling. Oncol. Lett. *12*, 3435–3440.
18. Yu, C., Zhang, B., Li, Y.L., and Yu, X.R. (2018). SIX1 reduces the expression of PTEN via activating PI3K/AKT signal to promote cell proliferation and tumorigenesis in osteosarcoma. Biomed. Pharmacother. *105*, 10–17.
19. Behbakht, K., Qamar, L., Aldridge, C.S., Coletta, R.D., Davidson, S.A., Thorburn, A., et al. (2007). Six1 overexpression in ovarian carcinoma causes resistance to TRAIL-mediated apoptosis and is associated with poor survival. Cancer Res. *67*, 3036–3042.
20. Du, P., Zhao, J., Wang, J., Liu, Y., Ren, H., Patel, R., Hu, C., Zhang, W., and Huang, G. (2017). Sine oculis homeobox homolog 1 regulates mitochondrial apoptosis pathway via caspase-7 in gastric cancer cells. J. Cancer *8*, 636–645.
21. Xie, Y., Jin, P., Sun, X., Jiao, T., Zhang, Y., Li, Y., and Sun, M. (2018). SIX1 is upregulated in gastric cancer and regulates proliferation and invasion by targeting the ERK pathway and promoting epithelial-mesenchymal transition. Cell Biochem. Funct. *36*, 413–419.
22. Liu, D., Li, L., Zhang, X.X., Wan, D.Y., Xi, B.X., Hu, Z., Ding, W.C., Zhu, D., Wang, X.L., Wang, W., et al. (2014). SIX1 promotes tumor lymphangiogenesis by coordinating TGFβ signals that increase expression of VEGF-C. Cancer Res. *74*, 5597–5607.
23. Coletta, R.D., Christensen, K., Reichenberger, K.J., Lamb, J., Micomonaco, D., Huang, L., Wolf, D.M., Müller-Tidow, C., Golub, T.R., Kawakami, K., and Ford, H.L. (2004). The Six1 homeoprotein stimulates tumorigenesis by reactivation of cyclin A1. Proc. Natl. Acad. Sci. USA *101*, 6478–6483.
24. Li, Z., Tian, T., Hu, X., Zhang, X., Nan, F., Chang, Y., Lv, F., and Zhang, M. (2013). Six1 mediates resistance to paclitaxel in breast cancer cells. Biochem. Biophys. Res. Commun. *441*, 538–543.
25. Chao, L., Liu, J., and Zhao, D. (2017). Increased Six1 expression is associated with poor prognosis in patients with osteosarcoma. Oncol. Lett. *13*, 2891–2896.
26. Zhang, X., and Xu, R. (2017). Six1 expression is associated with a poor prognosis in patients with glioma. Oncol. Lett. *13*, 1293–1298.
27. Nishimura, T., Tamaoki, M., Komatsuzaki, R., Oue, N., Taniguchi, H., Komatsu, M., Aoyagi, K., Minashi, K., Chiwaki, F., Shinohara, H., et al. (2017). SIX1 maintains tumor basal cells via transforming growth factor-β pathway and associates with poor prognosis in esophageal cancer. Cancer Sci. *108*, 216–225.
28. Yang, X., Zhao, H., Yang, J., Ma, Y., Liu, Z., Li, C., Wang, T., Yan, Z., and Du, N. (2019). miR-150-5p regulates melanoma proliferation, invasion and metastasis via SIX1-mediated Warburg effect. Biochem. Biophys. Res. Commun. *15*, 85–91.
29. Bartel, D.P. (2004). MicroRNAs: genomics, biogenesis, mechanism, and function. Cell *116*, 281–297.
30. Lai, E.C. (2002). Micro RNAs are complementary to 3′ UTR sequence motifs that mediate negative post-transcriptional regulation. Nat. Genet. *30*, 363–364.
31. Tong, A.W., and Nemunaitis, J. (2008). Modulation of miRNA activity in human cancer: a new paradigm for cancer gene therapy? Cancer Gene Ther. *15*, 341–355.

32. Mishra, P.J., Mishra, P.J., and Merlino, G. (2016). Integrated genomics identifies miR-32/MCL-1 pathway as a critical driver of melanomagenesis: implications for miR-replacement and combination therapy. PLoS ONE *11*, e0165102.

33. Chen, X., Wang, Y.W., Xing, A.Y., Xiang, S., Shi, D.B., Liu, L., Li, Y.X., and Gao, P. (2016). Suppression of SPIN1-mediated PI3K-Akt pathway by miR-489 increases chemosensitivity in breast cancer. J. Pathol. *239*, 459–472.

34. Gao, S., Liu, H., Hou, S., Wu, L., Yang, Z., Shen, J., Zhou, L., Zheng, S.S., and Jiang, B. (2018). miR-489 suppresses tumor growth and invasion by targeting HDAC7 in colorectal cancer. Clin. Transl. Oncol. *20*, 703–712.

35. Xie, Z., Cai, L., Li, R., Zheng, J., Wu, H., Yang, X., Li, H., and Wang, Z. (2015). Down-regulation of miR-489 contributes into NSCLC cell invasion through targeting SUZ12. Tumour Biol. *36*, 6497–6505.

36. Jiang, L., He, D., Yang, D., Chen, Z., Pan, Q., Mao, A., Cai, Y., Li, X., Xing, H., Shi, M., et al. (2014). miR-489 regulates chemoresistance in breast cancer via epithelial mesenchymal transition pathway. FEBS Lett. *588*, 2009–2015.

37. Patel, Y., Shah, N., Lee, J.S., Markoutsa, E., Jie, C., Liu, S., Botbyl, R., Reisman, D., Xu, P., and Chen, H. (2016). A novel double-negative feedback loop between miR-489 and the HER2-SHP2-MAPK signaling axis regulates breast cancer cell proliferation and tumor growth. Oncotarget *7*, 18295–18308.

38. Patel, Y., Soni, M., Awgulewitsch, A., Kern, M.J., Liu, S., Shah, N., Singh, U.P., and Chen, H. (2019). Correction: overexpression of miR-489 derails mammary hierarchy structure and inhibits HER2/neu-induced tumorigenesis. Oncogene *38*, 454.

39. Kuppa, S.S., Jia, W., Liu, S., Nguyen, H., Smyth, S.S., Mills, G.B., Dobbin, K.K., Hardman, W.J., and Murph, M.M. (2018). Autotaxin exacerbates tumor progression by enhancing MEK1 and overriding the function of miR-489-3p. Cancer Lett. *432*, 84–92.

40. Yuan, P., He, X.H., Rong, Y.F., Cao, J., Li, Y., Hu, Y.P., Liu, Y., Li, D., Lou, W., and Liu, M.F. (2017). KRAS/NF-κB/YY1/miR-489 signaling axis controls pancreatic cancer metastasis. Cancer Res. *77*, 100–111.

41. Kikkawa, N., Hanazawa, T., Fujimura, L., Nohata, N., Suzuki, H., Chazono, H., Sakurai, D., Horiguchi, S., Okamoto, Y., and Seki, N. (2010). *miR-489* is a tumour-suppressive miRNA target PTPN11 in hypopharyngeal squamous cell carcinoma (HSCC). Br. J. Cancer *103*, 877–884.

42. Zhang, L., Wang, Q., Wang, F., Zhang, X., Zhang, L., Tang, Y., and Wang, S. (2018). lncRNA LINC01446 promotes glioblastoma progression by modulating miR-489-3p/TPT1 axis. Biochem. Biophys. Res. Commun. *503*, 1484–1490.

OMTO, Volume *16*

Supplemental Information

miR-489-3p/SIX1 Axis Regulates Melanoma Proliferation and Glycolytic Potential

Xuhui Yang, Xiang Zhu, Zhifeng Yan, Chenxi Li, Hui Zhao, Luyuan Ma, Deyu Zhang, Juan Liu, Zihao Liu, Nan Du, Qinong Ye, and Xiaojie Xu

Figure S1

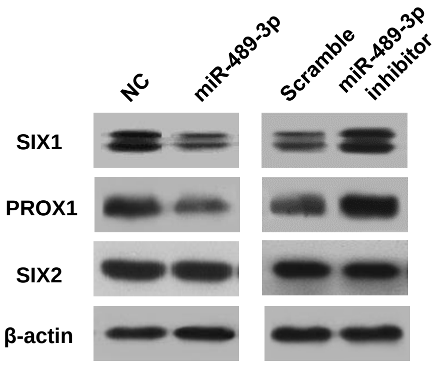

Supplementary Figure 1. Screening for the target of miR-489-3p in HEK293T cells. Immunoblot analysis of transfected with NC or miR-489-3p mimics, or scramble or miR-489-3p inhibitor in HEK293T cells . NC and scramble were used as negative control for miRNA mimics and miRNA inhibitor respectively. β–actin, a loading control.

Figure S2

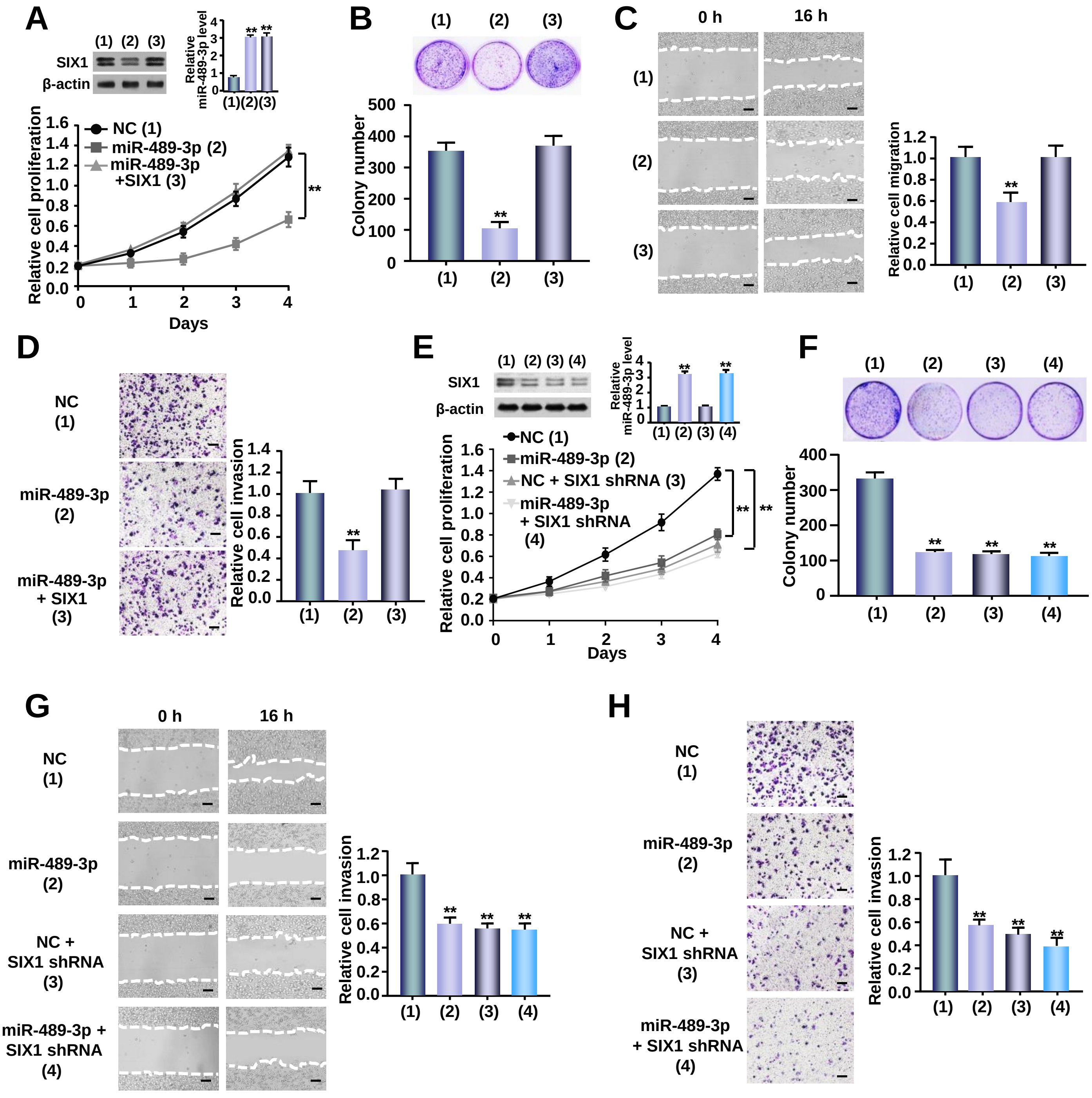

Supplementary Figure 2. miR-489-3p suppresses proliferation, migration and invasion via targeting SIX1 in SK-MEL-2 cells. (A) SK-MEL-2 cells were transfected with miR-489-3p mimics or miR-489-3p mimics plus SIX1 expression vector. Cell numbers was assessed by CCK-8 assay. The representative immunoblot shows SIX1 expression. Histograms display miR-489-3p expression determined by qRT-PCR. (B) Colony formation assay of SK-MEL-2 cells transfected as in (A). Illustrative images show colonies in plates (upper panels). Histograms show colony number. (C and D) Wound healing (C) and invasion (D) assays of SK-MEL-2 cells transfected as in (A). Right histograms show comparative cell migration and invasion. (E and F) Lentivirus mediated-SIX1 knockdown (SIX1 shRNA) or control SK-MEL-2 cells were transfected with NC or miR-489-3p mimics and analyzed as in (A) and (B). (G and H) Wound healing (G) and invasion (H) assays of lentivirus mediated-SIX1 knockdown (SIX1 shRNA) or control SK-MEL-2 cells were transfected as in (E). Scale bar, 100 μm. All values illustrated are mean ± SD of triplicate measurements and have been duplicated 3 times with similar results (***p* < 0.01 versus corresponding control).

Figure S3

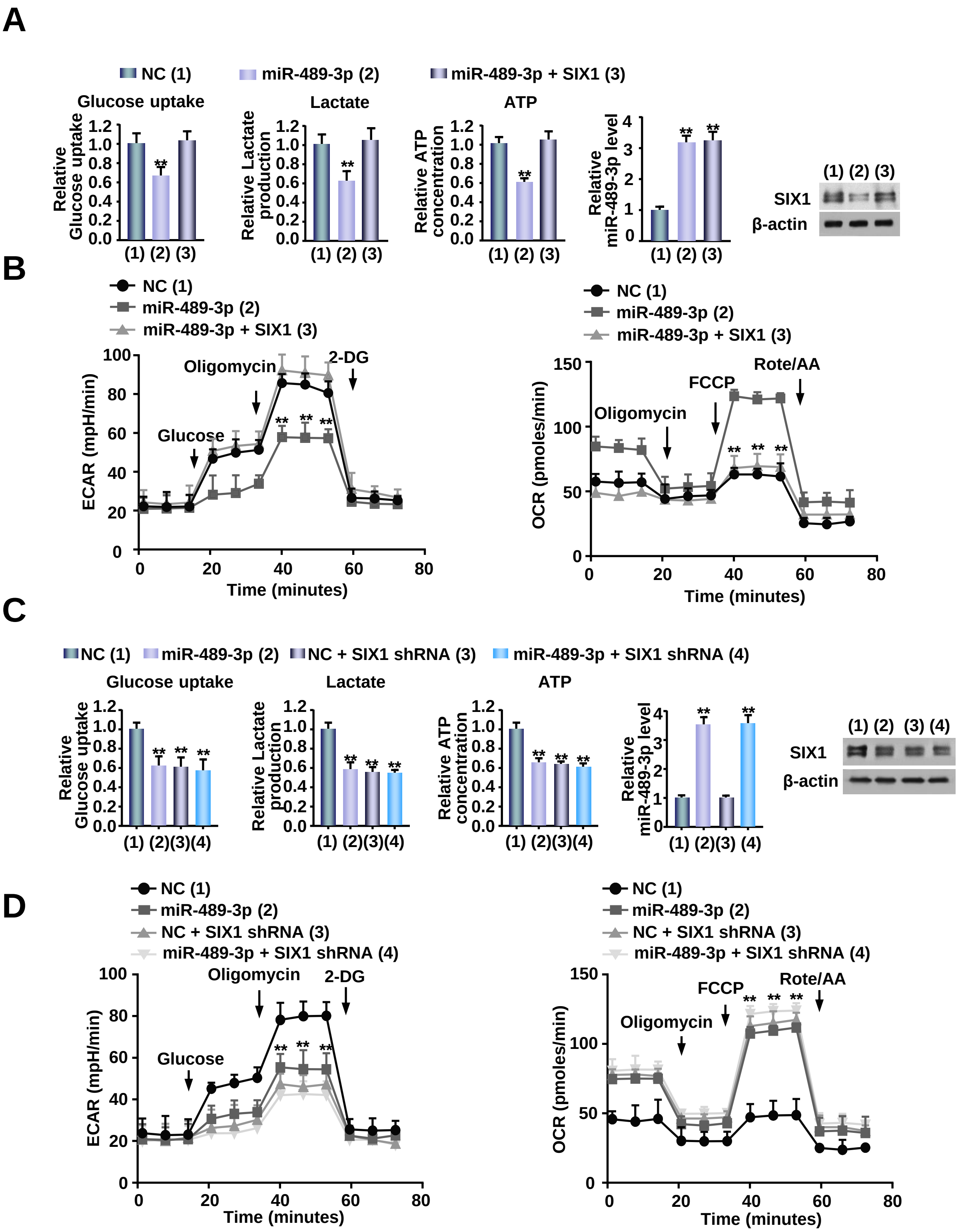

Supplementary Figure 3. miR-489-3p inhibits glycolysis via inhibition of SIX1 expression in SK-MEL-2 cells. (A) SK-MEL-2 cells were transfected with miR-489-3p mimics or miR-489-3p mimics plus SIX1. Glucose uptake and lactate production and ATP production were measured. Typical immunoblot reveals the expression of SIX1. qRT-PCR analysis shows miR-489-3p expression. (B) SK-MEL-2 cells were transfected as in (A), and ECAR and OCR were measured. (C) Lentivirus mediated-SIX1 knockdown (SIX1 shRNA) or control SK-MEL-2 cells were transfected with NC or miR-489-3p mimics and examined as in (A). (D) ECAR and OCR assays of lentivirus mediated-SIX1 knockdown (SIX1 shRNA) or control SK-MEL-2 cells were transfected as in (C). All values displayed are mean ± SD and have been duplicated 3 times with similar results (A-D). **$P < 0.01$ versus corresponding NC.

Figure S4

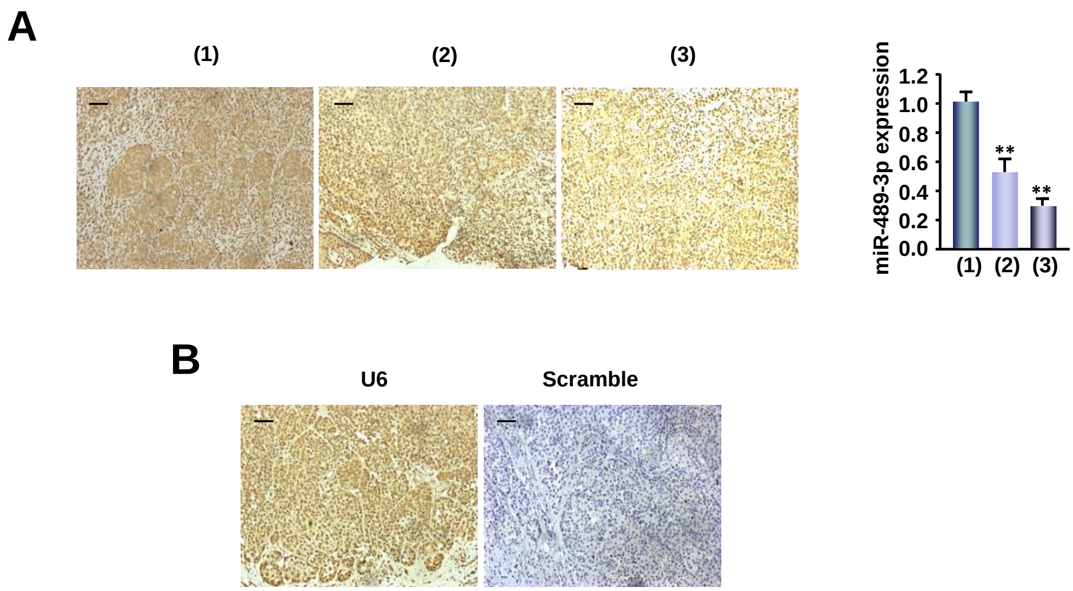

Supplementary Figure 4. Validation of the specificity of miR-489-3p probe. (A) Different miR-489-3p expression levels in 3 different melanoma tissues assessed by MISH (left panel) were measured by qRT-PCR (right panel). Data shown are mean ± SD of triplicate measurements and have been duplicated 3 times with similar results. ***P* < 0.01 versus case (1). (B) Positive control (U6) and negative control (Scramble) from the MISH kit were confirmed. Scale bar, 100 μm.

Figure S5

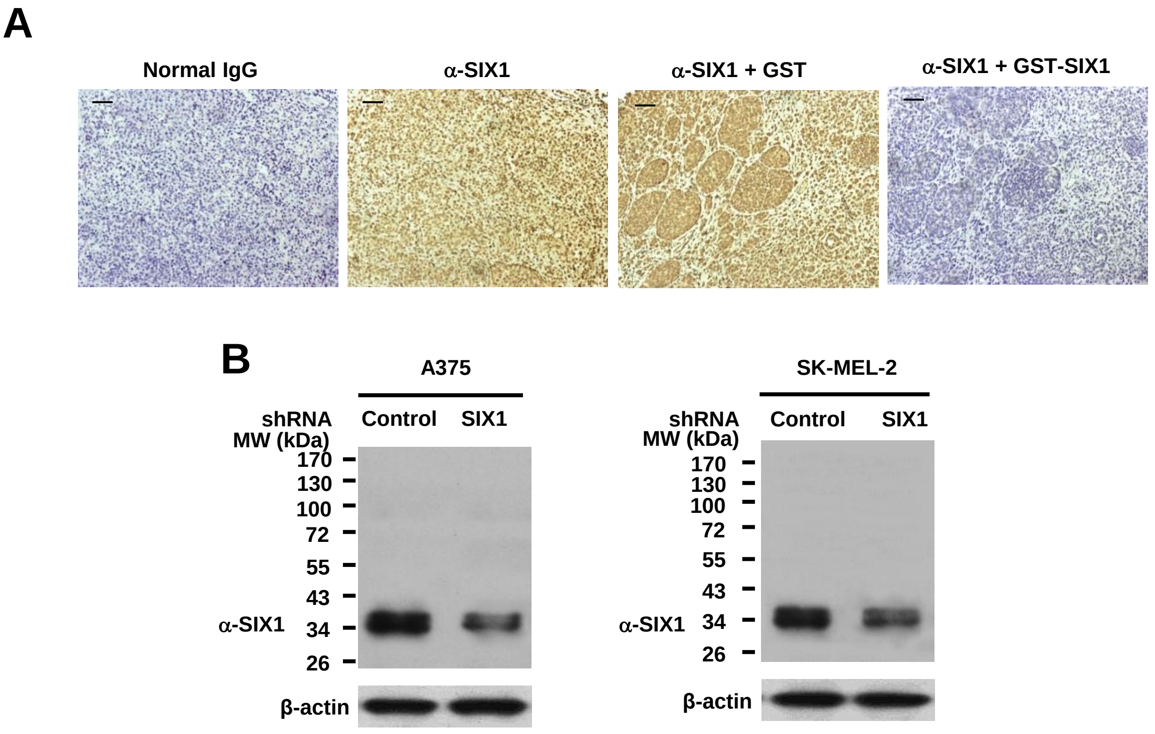

Supplementary Figure 5. Confirmation of antibody specificity to SIX1. (A) Immunohistochemical staining of melanoma samples incubated with normal IgG or anti-SIX1. To confirm antibody specificity, the anti-SIX1 was pre-incubated with recombinant GST-SIX1 protein or GST for 1 h prior to applying to tissue. Scale bar, 100 μm. (B) Immunoblot analysis of lysates from A375 (left panel) or SK-MEL-2 (right panel) cells infected with control shRNA or SIX1 shRNA using antibodies specific for anti-SIX1 β–actin, a loading control. MW, molecular weight.
